# Major changes in microbial diversity and community composition across gut sections of a juvenile *Panchlora* cockroach

**DOI:** 10.1371/journal.pone.0177189

**Published:** 2017-05-18

**Authors:** Erin A. Gontang, Frank O. Aylward, Camila Carlos, Tijana Glavina del Rio, Mansi Chovatia, Alison Fern, Chien-Chi Lo, Stephanie A. Malfatti, Susannah G. Tringe, Cameron R. Currie, Roberto Kolter

**Affiliations:** 1 Department of Microbiology and Immunobiology, Harvard Medical School, Boston, MA, United States of America; 2 Department of Bacteriology and Great Lakes Bioenergy Research Center, University of Wisconsin-Madison, Madison, WI, United States of America; 3 Department of Energy, Joint Genome Institute, Walnut Creek, CA, United States of America; Laurentian University, CANADA

## Abstract

Investigations of gut microbiomes have shed light on the diversity and genetic content of these communities, and helped shape our understanding of how host-associated microorganisms influence host physiology, behavior, and health. Despite the importance of gut microbes to metazoans, our understanding of the changes in diversity and composition across the alimentary tract, and the source of the resident community are limited. Here, using community metagenomics and 16S rRNA gene sequencing, we assess microbial community diversity and coding potential in the foregut, midgut, and hindgut of a juvenile *Panchlora* cockroach, which resides in the refuse piles of the leaf-cutter ant species *Atta colombica*. We found a significant shift in the microbial community structure and coding potential throughout the three gut sections of *Panchlora* sp., and through comparison with previously generated metagenomes of the cockroach’s food source and niche, we reveal that this shift in microbial community composition is influenced by the ecosystems in which *Panchlora* sp. occurs. While the foregut is composed of microbes that likely originate from the symbiotic fungus gardens of the ants, the midgut and hindgut are composed of a microbial community that is likely cockroach-specific. Analogous to mammalian systems, the midgut and hindgut appear to be dominated by Firmicutes and Bacteroidetes with the capacity for polysaccharide degradation, suggesting they may assist in the degradation of dietary plant material. Our work underscores the prominence of community changes throughout gut microbiomes and highlights ecological factors that underpin the structure and function of the symbiotic microbial communities of metazoans.

## Introduction

Over the past decade, there has been an increased appreciation for the fundamental roles that gut-microbiota play in the promotion of their host’s health and fitness. Members of the host gut microbiome have been shown to digest and make available nutrients and/or energy from otherwise recalcitrant dietary substrates, protect the host against pathogen invasion, and influence host behavior [1–3]. Interest in understanding gut microbiomes, coupled with advances in deep sequencing technology, [4–15] has provided invaluable insight into the composition and metabolic activities of animal gut microbial communities. Yet despite previous work, the ecological processes underpinning the composition and function of these symbiotic communities, as well as how microbial community composition and physiological potential changes across gut sections, is only poorly understood.

Community metagenomics and small subunit (SSU) rRNA gene surveys have provided detailed information regarding the phylogenetic composition of the microbiota inhabiting metazoan guts. The bacterial phyla that tend to dominate these microbial communities include Firmicutes, Bacteroidetes, Proteobacteria and Actinobacteria [8, 16–20], and the microbial communities of mammalian guts have generally been found to be more diverse than those of invertebrates [21]. However, some insects have been found to have highly diverse gut microbiota; cockroaches and termites in particular have been shown to harbor highly diverse microbial communities that help degrade recalcitrant material in the insect’s diet [10, 11, 14, 18, 22–29]. The composition of metazoan gut microbiota has been suggested to change considerably throughout the alimentary tract of the host, likely due to the distinct physical and chemical features of different gut sections, but few studies have investigated these changes directly.

Cockroaches are members of the insect order Blattodea, and thousands of species have been described worldwide [30]. Cockroaches are most often found in tropical or subtropical areas, where they play important roles in nutrient cycling [30]. Adult cockroaches of the genus *Panchlora* are pale green in color, and *Panchlora nivea*, the dominant species in the Caribbean, is found throughout Central and northern South America, as well as in North America, where it is often transported in banana shipments [31]. *Panchlora* cockroaches are also found in the rotting trunks of coconut and palm trees [31]. Juvenile *Panchlora* sp. are brown in color, and will burrow under logs, leaf litter and other debris [32].

**Fig 1:**
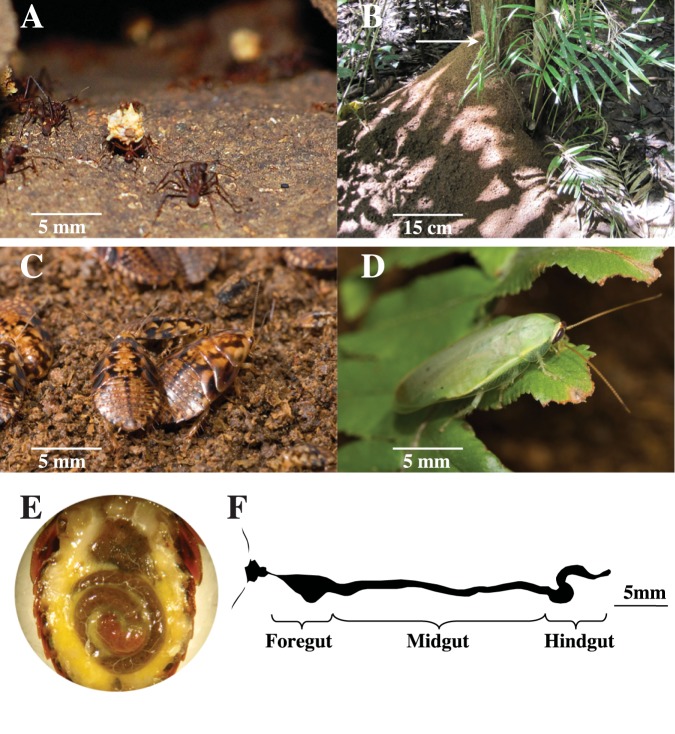
*Atta colombica* leaf-cutter ants remove waste material from their nest (A) and dispose of it in a single refuse pile on the rainforest floor (B). The most conspicuous insects in the leaf-cutter ant refuse pile were juvenile *Panchlora* cockroaches (C), which for this study were collected from the top 5 cm of the refuse pile (arrow in B). Adult *Panchlora* cockroaches (D) deposit young in the refuse pile and their young consume and thrive on the nutrient rich material, as evidenced by a refuse packed gut and enlarged fat body (E). Individually dissected guts were sectioned into the foregut, midgut, and hindgut (F). Photo credit: Robert Cullen (A), Erin A. Gontang (B/E), Justin C. Touchon (C/D).

Here, we focus on the gut microbiota of a juvenile *Panchlora* cockroach found living in and feeding on the refuse of the leaf-cutter ant species *Atta colombica* (Fig 1A). Leaf-cutter ants of the genus *Atta* are among the most conspicuous herbivores in the New World tropics, and derive nutrition from a specialized fungus they cultivate on collected foliar biomass [33, 34]. The agricultural practice of these ants generates massive quantities of garden waste material, including partially degraded plant material, the ant’s mutualistic fungus, and a diverse bacterial community [35–37], which *A*. *colombica* workers remove from the nest (Fig 1A) and deposit onto a large, external refuse pile (Fig 1B). The juvenile *Panchlora* cockroaches (Fig 1C) are one of the largest and most abundant insects within the *A*. *colombica* refuse piles in and around Gamboa, Panama. The adult *Panchlora* cockroach (Fig 1D) does not associate with leaf-cutter ants directly, but births live young in the *A*. *colombica* refuse piles. The immature cockroaches develop fully in this niche, feeding directly on the refuse material (Fig 1E). Culture-independent and metagenomic investigations of the *A*. *colombica* garden and refuse pile have previously been conducted [35–37], providing an opportunity to characterize and study the microbial community of an insect gut for which the microbial community composition of the surrounding environment is well defined.

Here, through analysis of high-resolution Illumina community metagenomes and SSU amplicon sequences, we investigate the phylogenetic and functional diversity of the microbial communities within the juvenile *Panchlora* foregut, midgut, and hindgut (Fig 1F). We characterize the total microbial community of the juvenile *Panchlora* cockroach, assess the microbial community’s capacity to degrade, utilize and recycle the refuse material consumed by the cockroach, and address two specific questions. First, by comparing the microbial communities of the foregut, midgut, and hindgut, we address to what extent the microbial diversity and community structure and function change along the length of cockroach alimentary tract. Second, by comparing the *Panchlora* gut sections, leaf-cutter ant gardens, and refuse piles, each a microbial ecosystem associated with the degradation and utilization of the same partially degraded leaf fragments originally harvested by the ants, we examine how environmental factors influence microbial community diversity and structure, and how interacting microbial ecosystems influence one another in a natural setting. The close proximity of the ant refuse pile (an example of a free-living microbial community), the leaf-cutter ant garden (an example of a host-associated, external digestion system [38, 39]), and the *Panchlora* gut (an example of a host-associated, internal digestion system) offers a unique opportunity to examine the dynamics of multiple interacting microbial ecosystems.

## Materials and methods

We acknowledge Ministerio de Ambiente de Panamá (MiAmbiente) and the Smithsonian Tropical Research Institute (STRI) for permission to collect material in the Republic of Panama.

### Cockroach collection and dissection

A total of 485 juvenile *Panchlora* cockroaches were collected from the external refuse piles of the leaf-cutter ant, *Atta colombica*, in Gamboa, Panama (latitude 9° 7’ 0” N, longitude 79° 42’ 0” W), from 7 to 22 November 2010. Only the largest juvenile cockroaches (1.2–1.4 cm) were collected from the top five cm of each refuse pile. Following collection, 275 of the cockroaches were kept in a breathable container with refuse pile material until they were dissected (within 4 hours). The other 210 cockroaches were immediately placed into a sterile 20% glycerol stock solution and kept frozen at -20°C until transported to the United States on dry ice. All cockroaches were dissected as described below.

Just prior to dissection, each of the 275 cockroaches were subdued (cooled for 5 minutes at -20°C) and then the head and protowings pinned. The legs and abdominal plate were removed with sterile spring scissors (Fine Science Tools Inc., Foster City, CA 91500–09) to expose the gut. Sterile forceps (Fine Science Tools Inc., Foster City, CA 91150–20) were used to grasp the esophagus and the rectum in order to remove the entire gut tract intact. Under sterile conditions, the foregut was separated from the midgut, the midgut from the hindgut, and the hindgut from the rectum (Fig 1F), prior to being placed into a sterile 20% glycerol stock solution. The gut sections were kept at -20°C until transport to the United States on dry ice. Upon arrival, samples were immediately placed at -80°C until nucleic acids extraction. The 210 cockroaches transported whole on dry ice to the United States were thawed in small batches and the guts dissected in an identical fashion to those dissected in the field.

Voucher specimens of the juvenile and adult *Panchlora* cockroaches were deposited in the Smithsonian Tropical Research Institute’s Insect Collection, in Ancon, Panama.

### Nucleic acid extraction

Total DNA was extracted in preparation for either 16S rRNA gene amplicon or community metagenomic sequencing. Total community DNA was extracted from 485 individual cockroach gut sections (eight separate extractions were performed on from 32 to 160 individual gut sections) and then pooled. By pooling DNA from the 485 cockroach gut sections, it was possible to isolate a sufficient amount of DNA for sequencing and to help ensure that the microbial community diversity and genomic potential associated with the *Panchlora* cockroach was well represented. Enrichment of the bacterial fraction was completed as follows. The foregut and hindgut sections were cut open lengthwise using scissors while submerged in PBS-1% tween-20. To be consistent in the amount of time taken to process gut section contents, and because the midgut is long and exceptionally thin, which supports nutrient absorption but makes a lengthwise cut difficult, the midgut sections were ground using a sterilized mortar and pestle. The gut sections were brought up in 35 mL PBS-1% tween-20, gently rocked for 10 minutes at room temperature, and then spun for 10 minutes at 40 x g. Twenty mL of supernatant were removed and the process repeated three additional times. The supernatants were pooled, the cells pelleted at 2,800 x g for 15 minutes, and then the pelleted cells were resuspended in 5 mL PBS-1% tween-20 and filtered through a 100 μm nylon filter (Millipore, Billerica, MA NY1H02500). The filtered cells were spun at 2,800 x g for 25 minutes and after the supernatant removed, an overnight extraction of the total DNA performed in 3 mL lysis buffer (20 mM Tris-HCl, pH 8.0, 2 mM sodium EDTA, 1.2% Triton X-100, 20 mg/mL lysozyme, 2 mg/mL RNase A).

Total DNA was purified using the Qiagen DNeasy Plant Maxi Kit (Qiagen, Valencia, CA) with the following modifications: cells were incubated at 65°C with buffer AP1 for 90 minutes and incubated on ice with buffer AP2 for 20 minutes. The DNeasy maxi spin columns were eluted twice with 750 μl of prewarmed elution buffer (10 mM Tris, 1 mM EDTA, pH 8.0). The eluant was pooled, combined with 1/10 volume sodium acetate and 3 times the volume of ethanol, and incubated on ice for 30 minutes. Following incubation, the DNA was added to spin columns from the DNeasy Blood and Tissue Kit (Qiagen, Valencia, CA), washed according to the manufacture’s recommendation and then eluted two times with 50 μl prewarmed elution buffer (10 mM Tris, 1 mM EDTA, pH 8.0). DNA concentration was determined by NanoDrop (Thermo Scientific, Wilmington, DE) and DNA quality checked by using gel electrophoresis with standards provided by the Joint Genome Institute (JGI).

### Metagenome sequencing, assembly, and annotation

Community metagenomes of the foregut, midgut, and hindgut were generated by using one full lane of Illumina for each gut section. Libraries for sequencing on the Illumina GAIIx and on HiSeq 2000 were generated using a modified version of the Illumina standard protocol using Illumina’s Truseq library kit, which does not require a PCR amplification step. Two micrograms of genomic DNA were used to generate each library. The DNA was sheared using a sonicator (Covaris Inc.) to generate fragments of 100–300 bp in length. The fragments were size selected by SPRI to ~ 200 bp. Selected fragments were end-repaired, A-tailed and then ligated with Illumina paired end sequencing adapters (Illumina Inc.). IAZX library was sequenced on Illumina GAIIx while IBAA and IBAC were sequenced on Illumina HiSeq 2000, all with paired-end 150 cycle reads. Reads were quality trimmed from both ends by quality of 10 and assembled with SOAPdenovo (http://soap.genomics.org.cn/soapdenovo.html) using kmer lengths ranging from 85 to 105 and flags “-R -d 1”. Resulting contigs were then dereplicated and merged using Newbler (for small contigs) and Minimus2 (for large contigs and contigs from Newbler; http://sourceforge.net/projects/amos/). Read depths were estimated based on read mapping with Burrows-Wheeler alignment (BWA). Between 27% and 58% of the reads were assembled into contigs, yielding between 33 and 765 Mb of assembled sequence data for each community metagenome. Sequencing statistics for the community metagenomes are presented in Table 1. Open reading frame (ORF) prediction for all metagenomes was done using a combination of MetaGene [40] and BLASTX [41]. ORFs were then annotated via the IMG/ER pipeline [42] and loaded into IMG/M (http://img.jgi.doe.gov/cgi-bin/m/main.cgi [43]). The *Panchlora* cockroach foregut, midgut, and hindgut community metagenomes can be found in the NCBI Short Read Archive under the accessions SRX208104, SRX1888534, and SRX2041330.

**Table 1:** Sequencing statistics of the juvenile *Panchlora* foregut, midgut, and hindgut community metagenomes.

	Foregut	Midgut	Hindgut
**Amount of raw sequence (Gbp)**	10.45	30.50	40.62
**Number of trimmed reads**	62,472,056	199,890,119	254,283,177
**Number of reads generating contigs**	16,980,348	116,703,965	121,933,388
**Percent reads as contigs**	27.2%	58.4%	47.9%
**Number of assembled contigs**	67,330	1,832,965	1,096,312
**Number of contigs greater than 800bp**	7,242	67,144	114,899
**Largest contig (kbp)**	33.9	408.3	408.3
**N50 contig size (bp)**	510	397	567
**N90 contig size (bp)**	263	274	276
**Protein coding genes**	52,729	1,419,166	1,161,369
**Size of assembled data (Mbp)**	33.2	765.3	600.3

Phylogenetic binning was performed on all community metagenome contigs greater than 800 bp in length using a combination of BLASTn [41] and PhymmBL [44], as previously described [45]. Both BLASTn and PhymmBL binning used a dataset comprising all complete bacterial and archaeal genomes available on NCBI as of 1 January 2012. Contigs having BLASTn hits with an E-value less than 10 e-10 were classified according to their best hit, while other sequences were classified according to the PhymmBL output if they were scored with confidence greater than 75%. Sequences not meeting these BLASTn or PhymmBL criteria were termed “unclassified”.

Genes were predicted from all contigs greater than 800 bp using MetaGene and the IMG/ER pipeline. The KEGG KAAS server [46] was used for inference of metabolic pathways. CAZymes were annotated by comparing all predicted proteins against a custom CAZy database [47] using BLASTp and cross-referencing hits with Pfam [48], as previously described [33].

A non-metric multidimensional scaling (nMDS) analysis was performed using the statistical software PAST (http://folk.uio.no/ohammer/past/), employing the Cosine similarity index based on the counts of Clusters of Orthologous Groups (COG). Counts of COGs were averaged by the number of genes found in each sample.

### Cockroach identification

A phylogenetic tree was constructed using the cytochrome oxidase I (COI) genes of the juvenile cockroach and 17 other COI sequences of insects belonging to the Blattodea. COI gene sequences were recovered from each of the assembled metagenomes (IMG Genome ID 3300000059–61) using the COI gene from *Panchlora nivea* (KU684412.1) as a query for BLASTn (E-value = 0.0). The coverage of these genes ranged from 190-482X. These sequences were aligned with the other COI sequences using ClustalX [49]. A complementary sequence from *Ancistrotermes cavithorax* (order Isoptera) (JF302843.1) was used as the outgroup. The phylogenetic tree was constructed using the maximum likelihood (ML) method. The phylogenetic distances were estimated with the Tamura-Nei [50] model using the MEGA 6.06 program [51]. Robustness of tree branches was determined by bootstrap analysis using 1000 repetitions. The *Panchlora* cockroach COI genes recovered from the foregut, midgut, and hindgut metagenomes can be found in NCBI under the GenBank accession numbers KY741985, KY741983 and KY741984, respectively.

To compare the mitochondrial DNA (mtDNA) of the juvenile cockroach to *Panchlora nivea*, a mtDNA scaffold was reconstructed using the *Panchlora nivea* complete genome as reference (KU684412.1). Metagenomic reads from the juvenile cockroach foregut sample were mapped to the reference genome using the software Geneious 10.0.9, allowing a maximum of 35% of mismatches to the reference and a maximum gap size of 5 bp. 74,276 reads were used to reconstruct a 16,201 bp consensus sequence. The average nucleotide identity between the resulting juvenile *Panchlora* scaffold and the *Panchlora nivea* complete genome was calculated using http://enve-omics.ce.gatech.edu/ani/.

### Pyrosequencing and 16S amplicons analyses

To generate amplicons, partial length 16S rRNA gene amplicon libraries of the V6-V8 region were constructed and sequenced as previously described [33] by using the same DNA samples submitted for community metagenomic sequencing of the foregut, midgut, and hindgut. 16S rRNA gene amplicon libraries that had been generated previously from leaf-cutter ant gardens [36] and refuse piles [52] were also analyzed for comparison. All 16S amplicons were quality checked, aligned, examined for the presence of chimeras, and clustered into 97% identity Operational Taxonomic Units (OTUs) using the program Pyrotagger [53].

The length of all 16S amplicons was trimmed to 250 bp using the online Pyrotagger interface before further processing. Representatives from each OTU were added to the Ribosomal Database Project's online Seqmatch tool (http://rdp.cme.msu.edu/seqmatch) [54] for classification. Only those matches having the highest Seqmatch score were used. The microbial community diversity of the individual gut sections was compared to previously sequenced microbial communities associated with leaf-cutter ant gardens and refuse piles by using UniFrac (http://bmf.colorado.edu/unifrac [55]) and performing both weighted and unweighted calculations. Amplicons were also processed with the program Mothur [56] using the 454 SOP [57]. OTUs were generated at 97%, 95%, and 90% identity in Mothur and OTU classification was performed by comparing representatives from each OTU to the NCBI 16S rRNA gene sequences (Bacteria and Archaea). All 16S amplicons can be found in the NCBI Short Read Archive under accession SRP105350.

## Results and discussion

### Community metagenome and 16S ribosomal gene amplicon libraries

**Fig 2:**
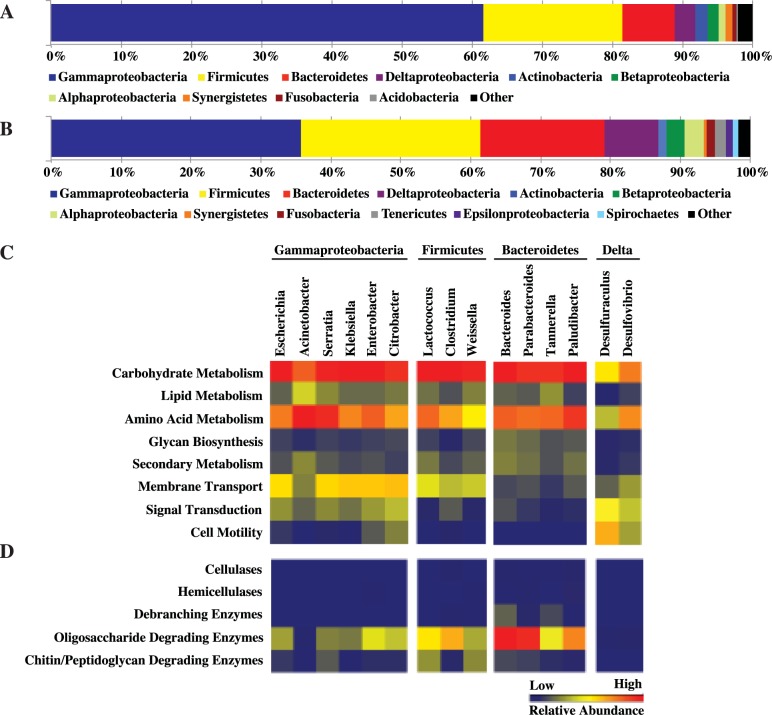
Phylum level classification of 16S rRNA gene amplicons (A) and metagenomic sequence data (B) for the juvenile *Panchlora* cockroach gut. Partial list of bacterial KEGG category (C) and CAZy (D) distribution for dominant bacterial community members (genera representing > 1% of the total metagenomic sequence); the heat maps illustrate the percent of the metagenomic sequence data annotated to the particular KEGG category (0–18%) or CAZy family grouping (0–0.014%), normalized by the total number of proteins binned to the genus. For the total bacterial KEGG category distribution for the foregut, midgut, and hindgut, and the total KEGG category and glycosyl hydrolase (GH) distribution of dominant bacterial community members, see S1 Fig.

Pyrosequencing of the bacterial 16S rRNA genes yielded 41,254 high quality reads (heretofore referred to as “16S amplicons”); 14,112, 12,906, and 14,236 from the foregut, midgut, and hindgut, respectively (S1 Table). Phylogenetic analyses of 16S amplicons from all gut sections indicated that Gammaproteobacteria, Firmicutes, and Bacteroidetes are the dominant bacteria within the juvenile *Panchlora* cockroach gut (Fig 2A). Of the 16S amplicons, 61.6% were classified as Gammaproteobacteria, 19.9% were classified as Firmicutes, and 7.3% were classified as Bacteroidetes (Fig 2A).

To more thoroughly assess the phylogenetic diversity and investigate the functional capacity of the *Panchlora* microbial community, deep Illumina sequencing of the juvenile cockroach gut was performed. Community metagenomic sequencing yielded over 81.5 Gbp of raw sequence data and over half a billion trimmed reads (Table 1). After reads were assembled into contigs and the contigs phylogenetically binned, it was determined that metagenomic sequencing recovered primarily bacterial sequences (97.7% of the total assembled and classified bp). Phylogenetic classification of the metagenomic contigs supported the results of our 16S amplicon analysis, further indicating that the microbial community of the combined *Panchlora* gut sections was composed predominantly of Gammaproteobacteria, Firmicutes, Bacteroidetes and Deltaproteobacteria (Fig 2B). While the overall pattern was similar to that described by the 16S amplicon analysis, fewer metagenomic sequences were classified as Gammaproteobacteria (35.1%), while a greater number of metagenomic sequences were classified as Firmicutes (25.4%), Bacteroidetes (17.4%), or Deltaproteobacteria (7.6%) (Fig 2B). Community sequencing of the *Panchlora* cockroach gut yielded a tremendous amount of metagenomic information and provided a less biased, more thorough analysis of the community composition.

Metagenomic sequencing also provided an opportunity to examine DNA sequences of the host cockroach. A single cockroach COI gene sequence was recovered from each of the assembled metagenomes and aligned with 17 other COI sequences of insects belonging to the Blattodea (S2 Fig). The recovery of a single COI gene sequence from each section, and the fact that the assembled COI sequences shared greater than 99% identity with one another, suggests the juvenile cockroaches represent a single species. The phylogenetic tree constructed using the maximum likelihood (ML) method suggests that the cockroaches are unlikely to be *Panchlora nivea*. Furthermore, using the *Panchlora nivea* mitochondrial genome as a scaffold (16,201 bp), we reconstructed a draft mtDNA genome, with an average coverage of 566X, for the juvenile *Panchlora* cockroach examined in our study. The juvenile *Panchlora* shared 89.0% nucleotide sequence identity to the *Panchlora nivea* sequence across the entire mitochondria.

In addition to phylogenetically binning the metagenomic sequences, the functional potential of the proteins encoded in the community metagenomic data was also analyzed (S2 Dataset). Almost 450,000 proteins were predicted, and of the roughly 40% that were annotated, the vast majority (73.3%) could also be taxonomically assigned to a bacterial genus. In order to assess the coding potential of the dominant bacterial groups, we analyzed the genus-level phylogenetic bins with over 1.5 Mbp of community metagenomic sequence data (Fig 2C). Dominant genera included members of the Gammaproteobacteria, Firmicutes, Bacteroidetes and Deltaproteobacteria, and while some variability in the overall coding potential was observed between phyla, the coding potential among dominant genera within the same phyla was similar (Fig 2C). Gammaproteobacteria harbored genes associated with carbohydrate and amino acid metabolism, as well as genes involved in membrane transport and signal transduction. Firmicutes and Bacteroidetes also harbored genes associated with carbohydrate and amino acid metabolism, suggesting that the dominant genera within these phyla and the Gammaproteobacteria may participate in the degradation of refuse consumed by the cockroach, material that includes partially degraded plant matter. Members of these phyla may also supplement the diet of the cockroach by providing essential amino acids. The dominant Deltaproteobacteria genera harbored a large percentage of genes associated with cell motility and signal transduction, suggesting these bacterial groups rapidly respond to changing conditions in the gut.

The refuse material removed from *A*. *colombica* nests is composed of partially degraded plant material, the mutualistic fungus cultured by the ants, and a diverse bacterial community [35, 36]. To investigate whether the dominant bacterial community members of the juvenile *Panchlora* gut assist in degrading the plant polymers associated with their diet, predicted proteins were compared to the carbohydrate active enzyme (CAZy) database [47] (Fig 2D). Debranching and oligosaccharide degrading enzymes were abundant in certain genera, especially within the Bacteroidetes genera *Bacteroides* and *Parabacteroides*, which were found to possess a large number of enzymes in glycosyl hydrolase (GH) families 2, 3, 13, 43, 78 and 92 (S3 Dataset). These groups also encoded several GH5 enzymes, which may be involved in the degradation of more recalcitrant polysaccharides, such as cellulose. While the Bacteroides harbored a large number of debranching and oligosaccharide degrading enzymes, the predominant Firmicutes, including *Lactococcus*, *Weissella* and *Clostridium*, generally lacked these enzymes (Fig 2D). While the Firmicutes may not be participating in plant biomass degradation, they did have GHs, including GH25 and GH73 (S3 Dataset), associated with the degradation of the bacterial cell wall component peptidoglycan. The analysis suggests that while Bacteroidetes may further degrade already partially digested leaf material consumed by their cockroach host, Firmicutes may utilize other bacteria as their primary carbon source, perhaps even bacteria consumed as part of the leaf-cutter ant garden material.

### Microbiome of the foregut, midgut, and hindgut

**Fig 3:**
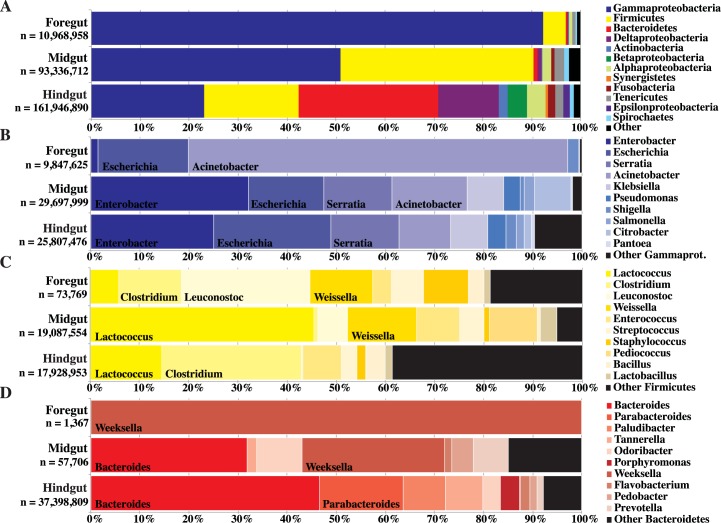
Phylum level classification of all assembled contigs (A) and the genus level classification for the Gammaproteobacteria (B), Firmicutes (C), and Bacteroidetes (D) of the *Panchlora* foregut, midgut, and hindgut; n is the total number of base pairs assembled and used to construct the graph. Genus level classification is shown for groups that make up more than 12% of each gut section. The metagenomic sequences provide evidence that microbial community composition changes and diversity increases along the alimentary tract.

To determine whether the microbial community of the *Panchlora* gut changes along the length of the alimentary tract, the metagenomes of the foregut, midgut, and hindgut were evaluated individually (Fig 3A). Gammaproteobacteria overwhelmingly dominated the foregut (91.8%), while in the midgut, the majority of sequences were assigned to either the Gammaproteobacteria or Firmicutes (49.8% and 38.5%, respectively). While both Gammaproteobacteria and Firmicutes were present in the hindgut (22.7% and 19.2%, respectively), the hindgut also harbored a prominent community of Bacteroidetes (28.1%). The relative abundance of the dominant phyla within the *Panchlora* sp. hindgut is similar to that found in the hindgut of a wild cockroach, *Blattella germanica* [28], which could indicate the existence of a core microbial community in the hindgut of omnivorous cockroaches, regardless of diet. In general, along the alimentary tract there was a clear shift in the community composition and an increase in the number of phyla observed (Fig 3A).

Analysis of the genus-level phylogenetic bins further established distinct community changes throughout the gut sections (Fig 3). Of the sequences that binned to the Gammaproteobacteria in the foregut, the vast majority belonged to either the genus *Acinetobacter* (78.2%) or *Escherichia* (18.7%) (Fig 3B). Within the midgut, although *Acinetobacter* and *Escherichia* populations were again prevalent (Fig 3B), the Firmicutes genus *Lactococcus* (45.4%) was dominant (Fig 3C). Within the hindgut, the amount of metagenomic sequence that binned to the Gammaproteobacteria, Firmicutes, and Bacteroidetes was similar (Fig 3A). Metagenomic sequences binning to the Bacteroidetes included *Bacteroides* (46.6%) and *Parabacteroides* (17.0%) (Fig 3D), while the Gammaproteobacteria sequences were most closely related to *Enterobacter* (33.4%) and *Escherichia* (31.8%) (Fig 3B), and the Firmicutes sequences were most closely related to *Clostridium* (28.3%) (Fig 3C). While not as abundant as the other phyla within the hindgut, Deltaproteobacteria made up 12.2% of the hindgut metagenome sequences, with sequences most closely related to the genera *Desulfovibrio* (60.5%) and *Desulfarculus* (13.2%) (S4 Dataset). Analysis of metagenomic sequences binned to the genus-level detailed how the community composition of the *Panchlora* cockroach gut changes between gut sections and revealed that community richness increases along the alimentary tract. The metagenomic sequence data also highlighted the presence of well-known anaerobic bacteria in the hindgut, suggesting that oxygen availability influences microbial community composition and contributes to the observed community changes.

Analysis of the 16S amplicon libraries generated from the different gut sections corroborate the metagenomic sequencing results, illustrating a clear increase in community richness along the alimentary tract. Comparison of rarefaction curves generated from the 16S amplicon libraries demonstrates that microbial diversity increases substantially throughout the *Panchlora* alimentary tract, with the hindgut being substantially more diverse than either the foregut or midgut (S3 Fig). While the hindgut was dominated by diverse Firmicutes genera, including *Lactococcus*, *Lactobacillus* and *Clostridium*, and Bacteroidetes genera, such as *Bacteroides* and *Parabacteroides*, the hindgut also harbored bacteria commonly found in the guts of ruminant mammals, including *Fusobacteria*, *Ruminococus*, *Megasphaera*, and *Butyrivibrio* [58–60]. While all minor members of the *Panchlora* hindgut, many of these genera (or related taxa) have been identified in other omnivorous cockroaches [18, 28, 29] and are likely among the diverse community members helping the cockroach to degrade and digest recalcitrant components of its food source. Analogous to mammalian gut systems, the cockroach gut appeared to select for low phylum level but high genus level diversity, and it is possible that low-abundance bacterial community members within dominant phyla help the cockroach to degrade lignocellulose present in the leaf-cutter ant garden refuse consumed.

Cockroaches are members of the insect superorder Dictyoptera, a group that also includes termites [61]. Previous culture-independent work revealed that the termite hindgut is dominated by cellulolytic genera, including *Spirochaeta* and *Fibrobacter* [14, 25], and cellulolytic *Spirochaeta* are also found in wood-feeding cockroaches [22]. While identified in termites and wood-feeding cockroaches, these genera were essentially absent from the juvenile *Panchlora* hindgut. No Fibrobacteria amplicons were identified in any *Panchlora* gut section, and only 13 Spirochaetia amplicons were identified in the hindgut. *Spirochaeta* and *Fibrobacter* DNA made up less than 0.2% of the hindgut metagenome sequence (S4 Dataset). Similarly, Spirochaetia and Fibrobacteria were not identified in 16S clone libraries of the omnivorous cockroach *Shelfordella lateralis* [29] yet were detected in very low abundance using a deep sequencing approach [18]. All together, the results suggest that the presence of *Spirochaeta* and *Fibrobacter*, especially *Spirochaeta*, correlates with a diet comprised primarily of wood. Other important members of the termite hindgut include Euryarchaeota [14, 62], including *Methanobrevibacter*, a microorganism capable of methanogenesis [63, 64]. While an important anaerobic process carried out in the termite hindgut, genes associated with methanogenesis were not abundant in the *Panchlora* hindgut and Euryarchaeota made up only 0.4% of the total metagenomic sequence (S4 Dataset).

Almost all cockroaches, as well as the primitive termite *Mastotermes darwiniensis*, harbor a bacterial endosymbiont in specialized cells within their abdominal fat body [65]. These endosymbionts, classified as a genus *Blattabacterium*, are purported to have co-evolved with their hosts for over 140 million years [66, 67], and are believed to provide benefit by playing a role in nitrogen waste recycling and nutrient provisioning [68]. Although the abdominal fat body is external to the cockroach gut, its close proximity made it impossible to avoid extracting bacterial DNA from the cockroach fat body. As such, metagenomic sequencing provided a sufficient number of reads to reconstruct the genome of the *Blattabacterium* endosymbiont residing within the juvenile *Panchlora* sp. (S4 Dataset). However, given *Blattabacterium* does not reside within the cockroach gut, all sequences associated with the endosymbiont were excluded from the present metagenomic analysis. In the future, a careful comparison of the *Panchlora* sp. endosymbiont with other *Blattabacterium* genomes will likely reveal how *Blattabacterium* contributes to the livelihood of the *Panchlora* sp. cockroach.

### Comparison of the foregut, midgut, and hindgut microbiomes with the leaf-cutter ant garden and refuse pile

**Fig 4:**
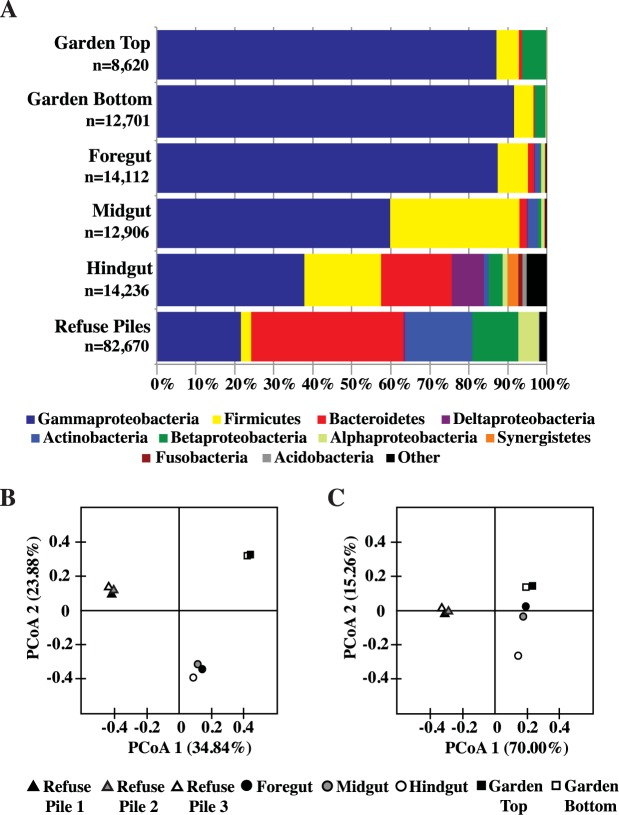
Class level classification of 16S rRNA gene amplicons for the *Panchlora* foregut, midgut, and hindgut, as compared to the *Atta colombica* garden top, garden bottom, and averaged refuse piles (A); n is the total number of 16S amplicons used to construct the graph. UniFrac unweighted principal coordinate analyses (PCoA) (B) and weighted PCoA (C) for the *Panchlora* foregut, midgut, and hindgut, and *A*. *colombica* garden top, garden bottom, and three different refuse piles. The 16S rRNA gene amplicons and weighted UniFrac analysis illustrate the shift in the *Panchlora* gut community composition along the alimentary tract and show that the cockroach gut communities are distinct from the *A*. *colombica* garden and refuse piles.

The microbial communities of the juvenile *Panchlora* cockroach gut, leaf-cutter ant garden, and the *Atta colombica* refuse pile all have access to the same partially degraded leaf fragments. In order to determine if the diversity of these microbial communities, each degrading the same nutrient source, varied depending on whether or not that community is associated with a host, we used 16S amplicons to compare the microbial communities associated with the refuse pile (an example of a free-living microbial community niche), the leaf-cutter ant garden (an example of a host-associated, external digestion niche [38, 39]), and the *Panchlora* gut (an example of a host-associated, internal digestion niche). Like the *Panchlora* cockroach foregut, the microbial community of the leaf-cutter ant garden [33, 36] was dominated by Gammaproteobacteria (Fig 4A). In contrast, the bacterial communities of the leaf-cutter ant garden and three cockroach gut sections were noticeably different from the bacterial community of the leaf-cutter ant refuse pile (Fig 4A). Given the high similarity of the leaf-cutter ant garden and juvenile *Panchlora* foregut bacterial communities, it appears that while the cockroaches live in the leaf-cutter ant refuse pile, they quickly sequester and feed almost exclusively on the most recently deposited waste material from the ant garden.

Even though the distribution of phyla present within the *Panchlora* foregut was almost identical to that of the leaf-cutter ant garden (Fig 4A), the dominant genera of the ant garden [36] varied from those dominant in the *Panchlora* foregut (Fig 2B) and gut as a whole (Fig 2A). To more rigorously compare the microbial community diversity of the *Panchlora* gut sections (foregut, midgut, and hindgut), the *A*. *colombica* garden (top and bottom), and the *A*. *colombica* refuse piles, unweighted and weighted UniFrac analyses of the 16S amplicons were performed. The unweighted UniFrac analysis clearly separated the microbial communities of the *Panchlora* gut sections, the *A*. *colombica* garden, and the *A*. *colombica* refuse piles (Fig 4B), and while the garden and refuse pile communities were tightly clustered, the *Panchlora* gut sections were more loosely clustered. The unweighted UniFrac analysis suggests that the microbial communities of the *Panchlora* gut sections, the *A*. *colombica* garden, and the *A*. *colombica* refuse piles are each distinct, and that while the microbial communities of the garden and refuse pile each shared highly similar community composition and membership, the microbial communities of the *Panchlora* gut sections were more distinct from one another. When sequence abundance was considered in addition to sequence diversity, the weighted UniFrac analysis clearly separated the *Panchlora* gut communities (Fig 4C), suggesting that the microbial community of each gut section is distinct. Of the *Panchlora* gut samples, the foregut community appeared to be most akin to the *A*. *colombica* garden communities, whereas the *Panchlora* hindgut community was noticeably different (Fig 4C). The weighted UniFrac analyses agree with the 16S amplicon results that suggest juvenile cockroaches consume the garden material most recently deposited on the refuse pile, and support the hypothesis that while microorganisms consumed by the cockroach may partially seed the microbial community of the cockroach foregut, the microbial community of each gut section is distinct. It is likely each *Panchlora* gut section provides a specialized niche, enriching for specific groups of bacteria.

While most of the Gammaproteobacteria genera present in the leaf-cutter ant garden [36] were also identified in the juvenile *Panchlora* foregut, the abundance of the Gammaproteobacteria genera varied between the two environments. For example, the dominant community member in the cockroach foregut, *Acinetobacter* (Fig 3B), is a minor member of the leaf-cutter ant garden microbial community, making up only 1.8% of the garden metagenomic sequence [36]. While less dramatic, the percent abundance of the second most prevalent genus, *Escherichia*, was up from 5.5% in the ant garden to 18.7% in the foregut. Comparison of the *Escherichia* population present in the *Panchlora* foregut to those residing in leaf-cutter ant fungus gardens revealed > 98% nucleotide identity for the majority of the binned contigs, strongly suggesting that the *Escherichia* population found in the ant gardens seeds the foregut community (S4 Fig). The *Acinetobacter* populations in these two environments were more distinct (S4 Fig), perhaps indicative of various species in the genus inhabiting both the ant gardens and the *Panchlora* foregut. Although it has been proposed that the foregut of cockroaches is unfavorable for microbial activity [69], conditions and the extended food residence time in the cockroach foregut [70] appear to favor and dramatically enrich for specific members of the Gammaproteobacteria.

**Fig 5:**
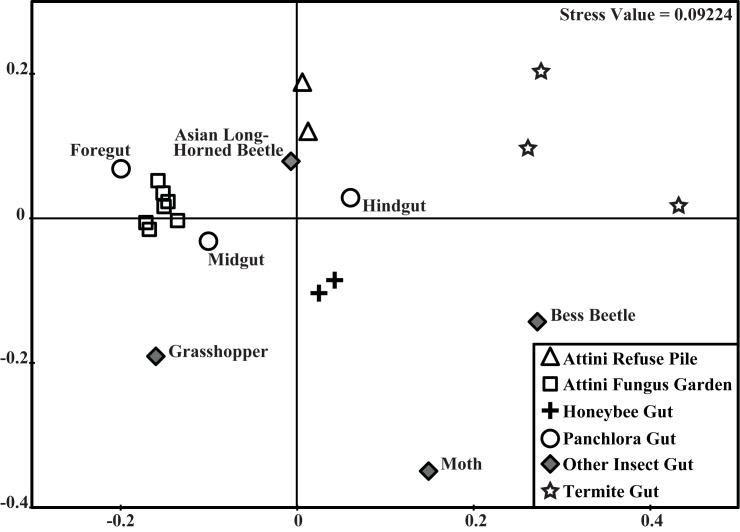
Non-metric multidimensional scaling (nMDS) based on the cosine similarity index of the Clusters of Orthologous Groups (COG) of 21 microbiomes representing the foregut, midgut, and hindgut of the *Panchlora* cockroach, the fungus gardens and refuse piles of Attini ants, and the guts of a grasshopper, a moth, beetles, honeybees, and termites. Metagenome information for each microbiome is available in the S5 Dataset.

To analyze the shift in functional potential throughout the *Panchlora* gut sections, as well as to assess the similarity of these microbiomes to leaf-cutter ant gardens, leaf-cutter ant refuse piles, and the microbial communities of other well-studied insect gut environments, we conducted a comparative analysis of the various microbiome’s functional potential. We compared three *Panchlora* gut sections, seven leaf-cutter ant fungus gardens, two leaf-cutter ant refuse piles and nine previously studied gut communities sampled from a grasshopper, a moth, an Asian long-horned beetle, a bess beetle, honeybees, and termites (Fig 5, S5 Dataset). Using Clusters of Orthologous Groups (COG) to assess functional potential, we found that the ant fungus garden communities cluster with the *Panchlora* foregut and midgut. The functional potential of the hindgut is clearly disparate from the other *Panchlora* gut sections, and no more similar to the foregut or midgut than to several other insect microbiomes. The results corroborate our phylogenetic analysis and comparison of the gut sections, and support our finding of a distinct shift from a fungus garden-like community in the foregut to a more canonical gut-like community in the hindgut.

While the bacterial community of the leaf-cutter ant garden appears to influence the community composition of the *Panchlora* foregut, the origin of many community members in the *Panchlora* hindgut remains unknown. One possibility is that the hindgut is seeded by bacteria consumed while living in the leaf-cutter ant refuse pile; however, it is equally possible the hindgut is seeded by cockroaches engaging in trophallaxis or coprophagy. It is well known that wood-feeding cockroaches and lower termites engage in proctodeal trophallaxis, the direct transfer of hindgut fluid from a donor to the mouth of a recipient, in order to transfer the hindgut microbiota, or digestive mutualists, required for the insect to survive on recalcitrant lignocellulose [71]. Juvenile *Panchlora* cockroaches may also engage in coprophagy [72]. The consumption of conspecific fecal material is highly likely given the abundance of voracious juveniles in the uppermost stratum of each refuse pile, and may be the leading contributor of bacteria that eventually populate the hindgut. Regardless, the high diversity and unique composition of the hindgut microbial community suggests a degree of host-specificity that necessitates a mechanism for transfer between individuals.

The 16S amplicons, coupled with the UniFrac analysis, suggested that the microbial communities of the *A*. *colombica* refuse pile, leaf-cutter ant garden, and *Panchlora* gut are all distinct from one another. The results indicated that even though each microbial community ultimately utilizes the same nutrient source, the microbial community’s environment affects and selects for a specific assemblage of bacteria. The bacterial community of the refuse pile, a non host-associated microbial niche exposed to the elements, was dominated by anaerobic and Gram-positive bacteria, including Actinobacteria [35] (S1 Dataset). The bacterial community of the leaf-cutter ant garden, a niche in which digestion of the material occurs external to the ant host in specialized underground chambers [38, 39], was dominated by Gammaproteobacteria, with members including *Enterobacter*, *Pantoea*, *Citrobacter*, *Klebsiella*, *Escherichia*, *and Acinetobacter* [36]. The host-associated microbial communities of the *Panchlora* foregut, midgut, and hindgut are each distinct, suggesting that both host-association, and the internal environment specific to each gut section, influences community composition.

## Conclusions

Metagenomic investigations of gut microbiomes shed light on the taxonomic diversity and genetic content of gut communities, providing the foundation to study how host-associated microorganisms influence host physiology, behavior and health. However, these studies are largely limited to vertebrate systems, and until now, metagenomic investigations of insect guts have focused primarily on insects with specialized diets, including the termite, corn borer and honeybee [5, 14, 73]. The microbial diversity and community structure associated with an omnivorous insect gut, and how that assemblage changes over the length of the alimentary tract, has not been well studied. In this study we characterized the microbial community composition of the *Panchlora* cockroach foregut, midgut, and hindgut, and compared these microbial communities to those of the already well defined and closely associated leaf-cutter ant fungus gardens and refuse piles. Our study reveals a distinct transition from the foregut community of this insect, which is composed primarily of bacterial genera present in the leaf-cutter ant fungus garden material used as a food source, to a host-specific microbiome in the midgut and hindgut composed primarily of Firmicutes and Bacteroidetes with a high capacity for lignocellulose degradation. Our work underscores the importance of the dynamic interplay between ecological interactions and host-specificity in shaping the complex microbial communities of metazoans.

## Supporting information

S1 Dataset Spreadsheet with 16S rRNA gene amplicon details. (XLSX)

S2 Dataset Spreadsheet with protein predictions/annotations of metagenomic sequence data (KEGG) details. (XLSX)

S3 Dataset Spreadsheet with protein predictions/annotations of metagenomic sequence data (CAZy) details. (XLSX)

S4 Dataset Spreadsheet with taxonomic classification of metagenomic sequence data (PhymmBL) details. (XLSX)

S5 Dataset Spreadsheet detailing the 21 metagenomes used for the non-metric multidimensional scaling (nMDS) of the Clusters of Orthologous Groups (COG). (XLSX)

S1 Fig Total bacterial KEGG category distribution for the foregut, midgut, and hindgut (A); n is the total number predicted proteins. Each sector represents the percentage of proteins annotated to a specific KEGG category (see legend).
Total KEGG category and glycosyl hydrolase (GH) distribution of dominant bacterial community members (genera representing > 1% of the total metagenomic sequence in one or more gut sections) (B). Left of the bar graph is the abundance (in percent) of each genus within the foregut (F), midgut (M) and hindgut (H), according to phylogenetic binning of the metagenomic data (gray when < 0.2%). Each color block represents the percentage of proteins annotated to a specific KEGG category or GH (see legend). Dominant genera included members of the Gammaproteobacteria, Firmicutes, Bacteroidetes and Deltaproteobacteria.
(PDF)

S2 Fig Phylogenetic tree based on the analysis of 576 bp of 21 COI gene sequences (NCBI accession numbers in parentheses), constructed using the maximum likelihood method (ML) in MEGA 6.06 with the Tamura-Nei model. Bootstrap values are indicated at each node. The scale bar represents evolutionary changes.
(PDF)

S3 Fig Rarefaction curves of the 16S rRNA gene amplicon libraries constructed for the foregut, midgut, and hindgut samples. OTU: Operational Taxonomic Unit. (PDF)

S4 Fig Histograms showing the percent identity of the best BLASTn hits recovered in a comparison of the contigs phylogenetically binned to the genera *Acinetobacter* and *Escherichia* in metagenomic data from the fungus gardens of *Atta colombica* leaf-cutter ants and the *Panchlora* sp. foregut. (PDF)

S1 Table Partial list of the Carbohydrate-Active enZymes (CAZy) in the *Panchlora* foregut, midgut, and hindgut, as compared to the *Atta colombica* garden top and bottom, wallaby foregut and termite hindgut. (PDF)
