# Supplementary figures and images for: Major changes in microbial diversity and community composition across gut sections of a juvenile Panchlora cockroach
Source: PLoS One. 2017 May 18;12(5):e0177189. doi: 10.1371/journal.pone.0177189 (PMC5436645; doi:10.1371/journal.pone.0177189)

S1 Figure

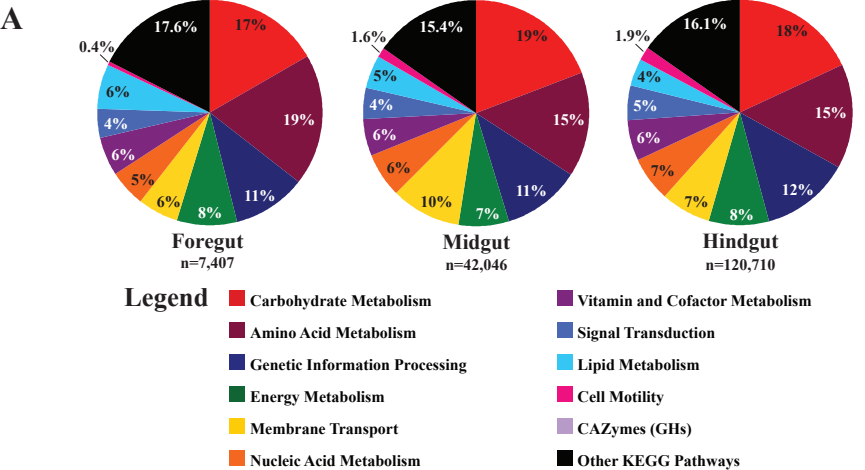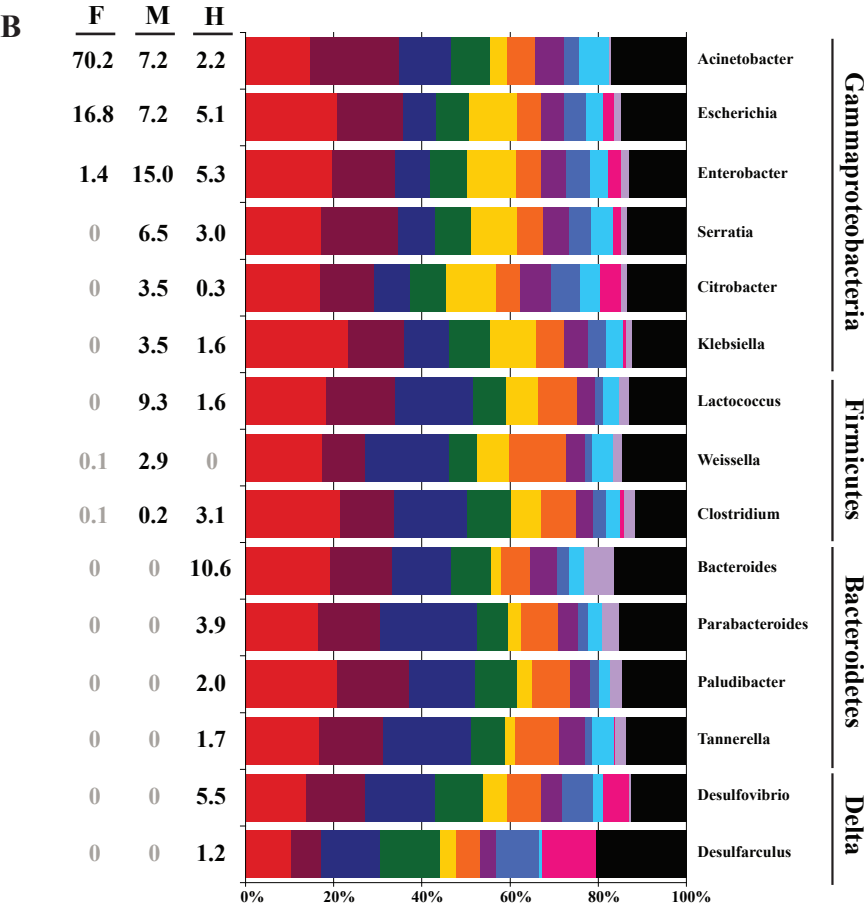

Supplement: S1 Fig — Total bacterial KEGG category distribution for the foregut, midgut, and hindgut (A); n is the total number predicted proteins. Each sector represents the percentage of proteins annotated to a specific KEGG category (see legend). Total KEGG category and glycosyl hydrolase (GH) distribution of dominant bacterial community members (genera representing > 1% of the total metagenomic sequence in one or more gut sections) (B). Left of the bar graph is the abundance (in percent) of each genus within the foregut (F), midgut (M) and hindgut (H), according to phylogenetic binning of the metagenomic data (gray when < 0.2%). Each color block represents the percentage of proteins annotated to a specific KEGG category or GH (see legend). Dominant genera included members of the Gammaproteobacteria, Firmicutes, Bacteroidetes and Deltaproteobacteria. (PDF) [file pone.0177189.s006.pdf]

S2 Figure

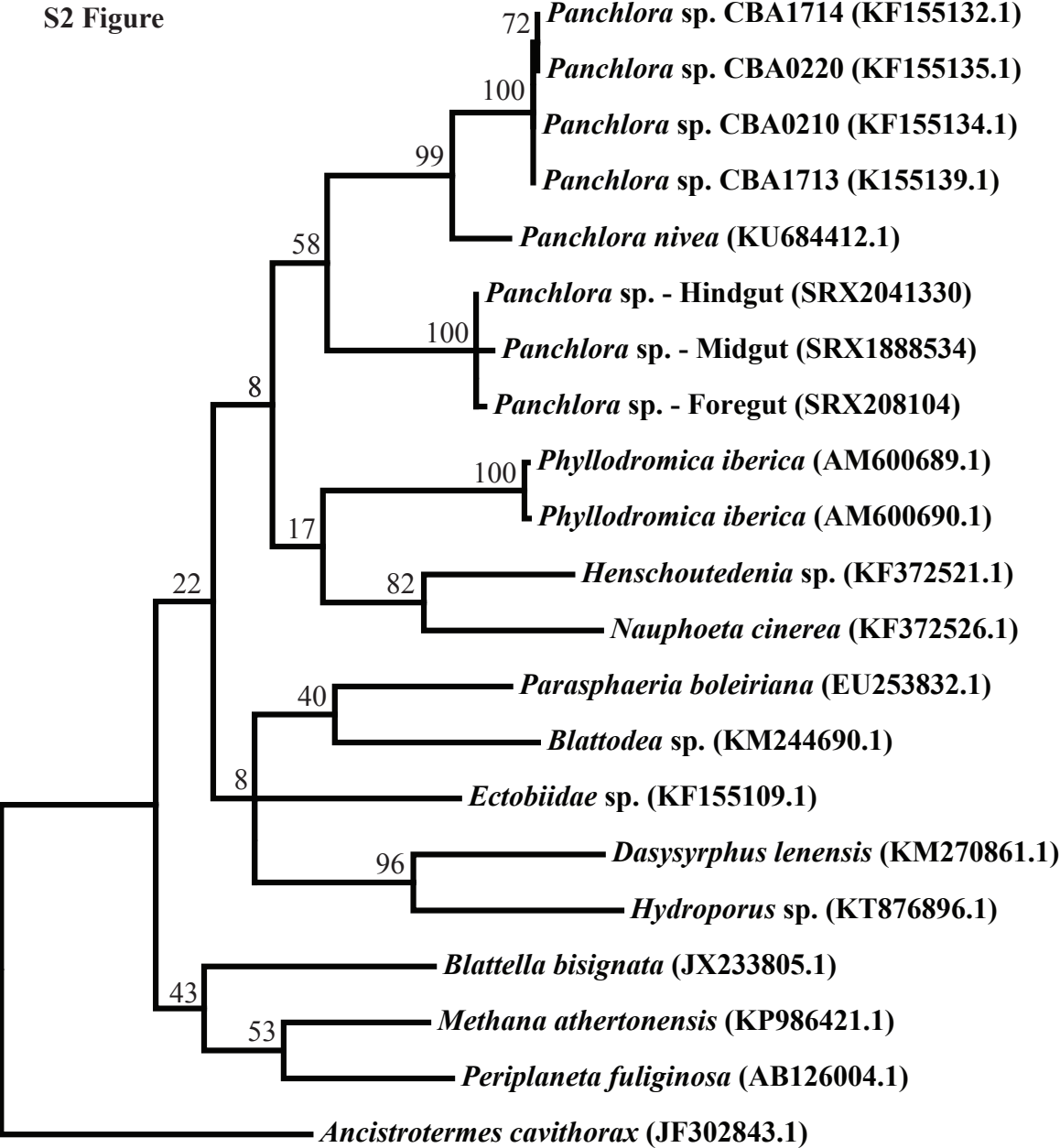

0.05

Supplement: S2 Fig — Bootstrap values are indicated at each node. The scale bar represents evolutionary changes. (PDF) [file pone.0177189.s007.pdf]

S3 Figure

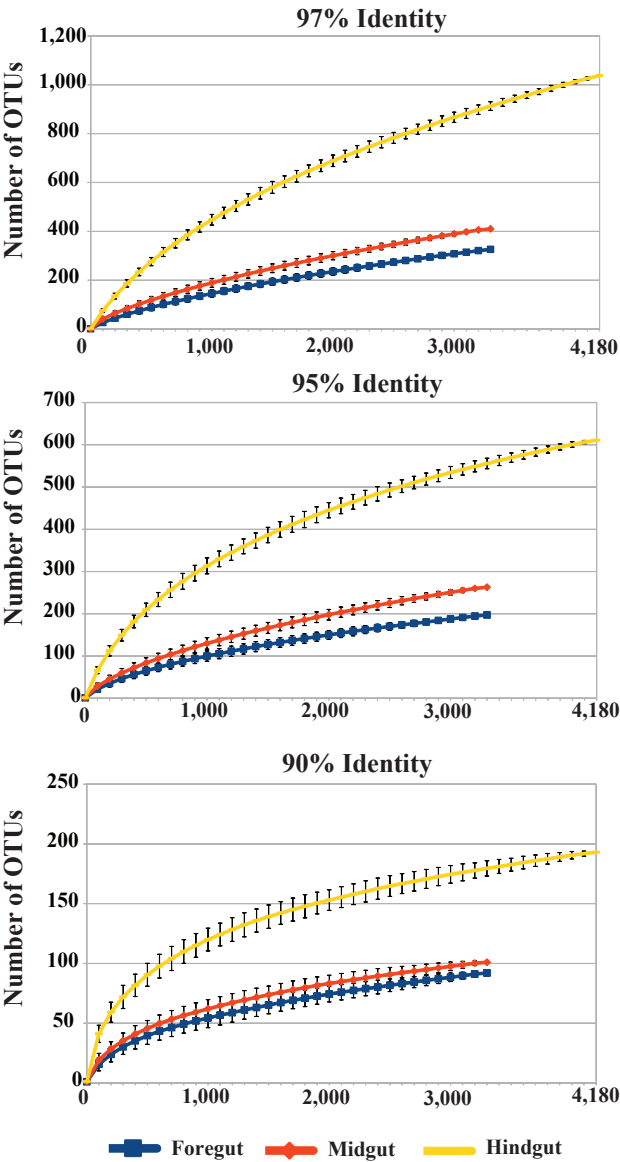

Supplement: S3 Fig — (PDF) [file pone.0177189.s008.pdf]

S4 Figure

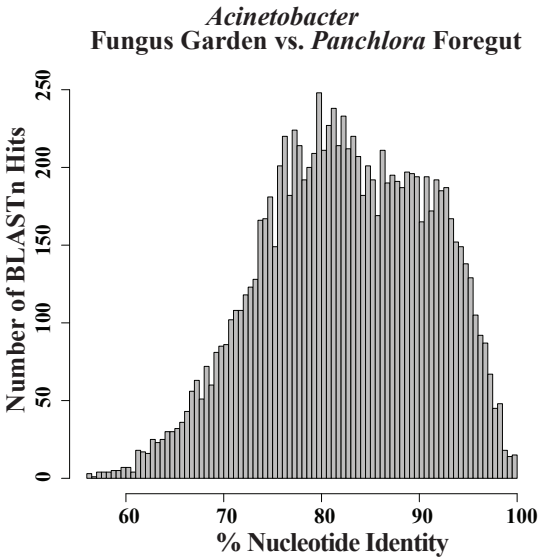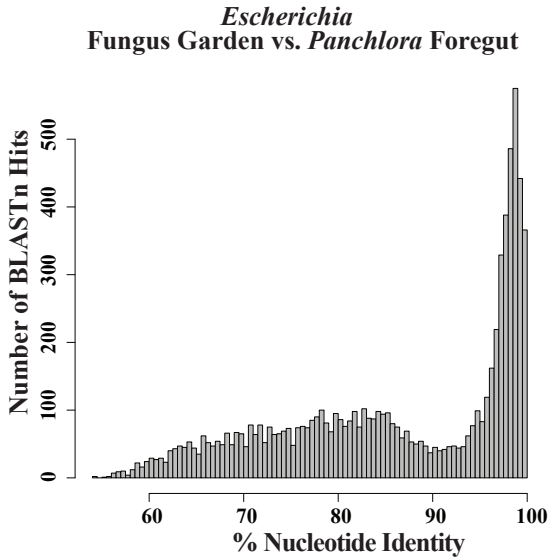

Supplement: S4 Fig — (PDF) [file pone.0177189.s009.pdf]
