# Supplementary material for: Major changes in microbial diversity and community composition across gut sections of a juvenile Panchlora cockroach
Source: PLoS One. 2017 May 18;12(5):e0177189. doi: 10.1371/journal.pone.0177189 (PMC5436645; doi:10.1371/journal.pone.0177189)
Supplement: S1 Table — (PDF) [file pone.0177189.s010.pdf]

S1 Table

	Foregut	Midgut	Hindgut	Garden Top	Garden Bottom	Wallaby	Termite	Known Activity
Cellulases								
GH5	0	1	75	5	2	20	97	Cellulase, mannosidase
GH6	0	0	0	0	0	0	0	Endocellulase, cellobiohydrolase
GH9	0	9	27	0	2	4	39	Endocellulase, cellobiohydrolase
GH44	0	0	18	0	0	0	4	Endoglucanase, xyloglucanase
GH45	0	0	0	0	0	0	6	Endoglucanase
Total	0 (0%)	10 (0.4%)	120 (1.5%)	5 (0.3%)	4 (0.2%)	24 (2.3%)	146 (10.1%)	
Hemicellulases								
GH8	3	50	26	36	30	2	17	Cellulase, xylanase, chitosanase
GH10	0	0	57	0	1	18	92	Xylanase
GH11	0	0	1	0	0	0	18	Xylanase
GH26	0	0	27	1	0	8	19	Xylanase, mannanase
GH53	0	0	24	1	4	3	5	Endogalactanase
Total	3 (1.9%)	50 (2.2%)	135 (1.7%)	38 (1.9%)	35 (2.1%)	31 (3.0%)	151 (10.4%)	
Debranching Enzymes								
GH51	0	3	15	4	2	18	26	Arabinofuranosidase
GH67	0	0	6	2	0	0	6	Glucuronidase
GH78	0	8	429	25	14	52	7	Rhamnosidases
GH105	0	0	76	25	3	1	2	Rhamnogalacturonyl hydrolase
Total	0 (0%)	11 (0.5%)	526 (6.6%)	56 (2.9%)	19 (1.2%)	71 (6.9%)	41 (2.8%)	
Oligosaccharide Degrading Enzymes								
GH1	16	531	658	256	243	84	27	Glucosidase, galactosidase, mannosidase
GH2	2	35	540	23	22	33	30	Galactosidase, mannosidase, glucuronidase
GH3	6	69	543	95	76	98	108	Glucosidase, xylosidase
GH4	7	81	139	61	55	3	17	Galactosidase, glucosidase
GH13	18	245	471	175	41	5	4	Glucosidase
GH28	0	6	235	13	11	10	13	Polygalacturonase
GH29	1	9	299	20	4	5	12	Fucosidase
GH31	4	89	338	74	71	21	4	Glucosidase, xylosidase
GH35	0	19	54	11	1	10	6	Galactosidase, glucosaminidase
GH36	0	11	34	26	24	29	4	Galactosidase, N-acetylgalactosaminidase
GH38	4	29	59	21	11	3	26	Mannosidase
GH39	0	8	37	1	0	3	11	Xylosidase, iduronidase
GH42	1	40	137	13	14	17	34	Galactosidase
GH43	4	31	231	39	36	3	57	Arabinase, xylosidase
GH52	0	0	0	0	0	0	3	Xylosidase
GH57	0	1	85	0	0	0	0	Galactosidase
GH92	0	0	81	16	1	1	17	Mannosidase
Total	63 (40.4%)	1204 (53.1%)	3941 (49.5%)	844 (43.1%)	610 (37.1%)	325 (31.6%)	373 (25.7%)	
Chitin and Peptidoglycan Degrading Enzymes								
GH18	2	74	98	52	20	6	30	Chitinase, N-acetylglucosaminidase
GH23	5	37	31	158	3	6	19	Lysozyme
GH24	1	27	44	29	11	31	4	Lysozyme
GH25	5	56	79	9	4	8	13	Lysozyme
GH20	0	26	211	31	5	8	4	N-acetylglucosaminidase
GH73	6	118	162	62	13	5	7	N-acetylglucosaminidase
Total	19 (12.2%)	338 (14.9%)	625 (7.9%)	341 (17.4%)	56 (3.4%)	64 (6.2%)	77 (5.3%)	
GH in Chart	85	1613	5347	1284	724	515	788	
% GH in Chart	82.5%	84.4%	82.8%	79.8%	63.2%	56.8%	64.1%	
Total GH	103	1912	6460	1610	1146	906	1230	
Total CAZy	156	2268	7956	1960	1643	1029	1452	
Total Protein	46501	867263	1124056	240966	199019	79862	82789	
% ORFs	0.34%	0.26%	0.71%	0.81%	0.83%	1.29%	1.75%	
